# Diffusion-prepared dark blood delayed enhancement imaging for improved detection of subendocardial infarcts

**DOI:** 10.1186/1532-429X-11-S1-O10

**Published:** 2009-01-28

**Authors:** Michael Salerno, Frederick H Epstein, Christopher M Kramer

**Affiliations:** University of Virginia, Charlottesville, VA USA

**Keywords:** Blood Pool, Inversion Recovery Sequence, Motion Sensitization, Vessel Wall Image, Delay Enhancement Image

## Introduction

Delayed enhancement MRI enables detection of non-viable myocardium with high spatial resolution and has become the gold standard for imaging myocardial infarction. However, subendocardial infarcts are sometimes difficult to detect as they may demonstrate similar image intensity as the ventricular cavity. A double inversion dark-blood pulse sequence to create black blood delayed enhancement images of myocardial infarction has been previously described [1–3]. The sequence relies on precise timing of non-selective and selective inversion pulses, and it is sensitive to incomplete exchange of blood and changes in T1 relaxation of the blood and myocardium. The technique significantly improves blood-infarct contrast at the expense of some reduction in SNR and infarct-myocardial contrast. Diffusion preparation-gradients have been used to create dark-blood vessel wall images [4], but have never been applied post-contrast for infarct imaging.

## Purpose

To develop a dark-blood delayed enhancement pulse sequence based on diffusion preparation which would not rely on complete blood exchange and would be relatively insensitive to changes in relaxation times.

## Methods

A diffusion-prepared inversion recovery (IR) pulse sequence was developed by adding a driven equilibrium module using a BIR-4 0 degree radiofrequency pulse with motion sensitization gradients between its components prior to a segmented FLASH readout. The timing of this preparation was optimized to minimize the effects of strain-induced signal loss. The technique was tested in a canine model of chronic infarction using a 1.5 T MR scanner (Magnetom Avanto, Siemens Medical Solutions). Images were obtained 5–10 minutes after injection of 0.15 mg/kg of Magnevist. Sequence parameters included field of view 300 mm, matrix 192 × 114, TE 2.7 ms, spatial resolution 1.6 × 2.3 × 10 mm, lines per segment 12, bandwidth 400 Hz/pixel, acquisition duration 16 heartbeats, effective b-value 0.25 s/mm^2^.

## Results

**Figure 1:**
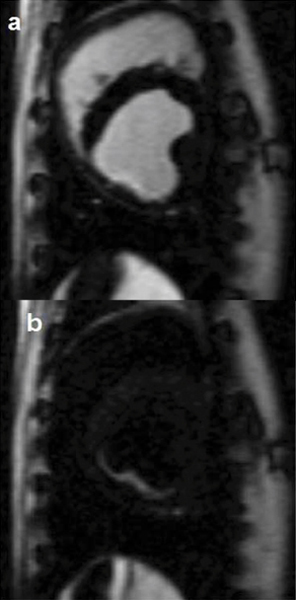
Figure 1

Figure 1(a) shows a standard bright-blood IR-FLASH delayed enhancement image. There is an infarct in the inferior wall which is difficult to distinguish from the blood pool. Figure 1(b) displays an image of the same slice that demonstrates the utility of the diffusion-prepared IR sequence. Here, the diffusion preparation causes suppression of the blood pool, improving the ability to detect the subendocardial region of infarction.

## Conclusion

We have developed a new dark-blood delayed enhancement pulse sequence which attenuates the blood pool based on motion sensitization. In preliminary studies, this technique improves delineation of subendocardial infarcts.
